# Impaired placental autophagy in placental malaria

**DOI:** 10.1371/journal.pone.0187291

**Published:** 2017-11-10

**Authors:** Kris Genelyn Dimasuay, Lan Gong, Fredrick Rosario, Emma McBryde, Tim Spelman, Jocelyn Glazier, Stephen J. Rogerson, James G. Beeson, Thomas Jansson, Rodney J. Devenish, Philippe Boeuf

**Affiliations:** 1 Burnet Institute, Melbourne, Victoria, Australia; 2 Department of Medicine at the Peter Doherty Institute, University of Melbourne, Parkville, Victoria, Australia; 3 Biomedicine Discovery Institute and the Department of Biochemistry and Molecular Biology, Monash University, Clayton, Victoria, Australia; 4 Department of Obstetrics & Gynecology, University of Colorado Anschutz Medical Campus, Aurora, Colorado, United States of America; 5 Victorian Infectious Diseases Service, Royal Melbourne Hospital, Parkville, Victoria, Australia; 6 Maternal and Fetal Health Research Centre, Faculty of Biology, Medicine and Health, University of Manchester, St. Mary’s Hospital, Manchester, United Kingdom; Institut de recherche pour le developpement, FRANCE

## Abstract

**Background:**

Placental malaria is a major cause of low birthweight, principally due to impaired fetal growth. Intervillositis, a local inflammatory response to placental malaria, is central to the pathogenesis of poor fetal growth as it impairs transplacental amino acid transport. Given the link between inflammation and autophagy, we investigated whether placental malaria-associated intervillositis increased placental autophagy as a potential mechanism in impaired fetal growth.

**Methods:**

We examined placental biopsies collected after delivery from uninfected women (n = 17) and from women with *Plasmodium falciparum* infection with (n = 14) and without (n = 7) intervillositis. Western blotting and immunofluorescence staining coupled with advanced image analysis were used to quantify the expression of autophagic markers (LC3-II, LC3-I, Rab7, ATG4B and p62) and the density of autophagosomes (LC3-positive puncta) and lysosomes (LAMP1-positive puncta).

**Results:**

Placental malaria with intervillositis was associated with higher LC3-II:LC3-I ratio, suggesting increased autophagosome formation. We found higher density of autophagosomes and lysosomes in the syncytiotrophoblast of malaria-infected placentas with intervillositis. However, there appear to be no biologically relevant increase in LC3B/LAMP1 colocalization and expression of Rab7, a molecule involved in autophagosome/lysosome fusion, was lower in placental malaria with intervillositis, indicating a block in the later stage of autophagy. ATG4B and p62 expression showed no significant difference across histological groups suggesting normal autophagosome maturation and loading of cargo proteins into autophagosomes. The density of autophagosomes and lysosomes in the syncytiotrophoblast was negatively correlated with placental amino acid uptake.

**Conclusions:**

Placental malaria-associated intervillositis is associated with dysregulated autophagy that may impair transplacental amino acid transport, possibly contributing to poor fetal growth.

## Introduction

Low birthweight (LBW), defined as a birthweight of a live infant weighing less than 2,500 grams, is a major global health issue affecting 16% of deliveries globally [1]. LBW is the biggest risk factor for more than 80% of neonatal deaths [2]. The WHO reaffirmed reducing the incidence of LBW as an important target of the UN Millennium Development Goal for reducing child mortality [1]. However, the mechanisms causing LBW in countries with the highest prevalence are poorly understood.

Malaria in pregnancy is one of the leading causes of LBW responsible for up to 900,000 LBW deliveries [3] and over 100,000 infant deaths annually in Africa alone [4]. Malaria in pregnancy can lead to placental malaria, defined as the sequestration of *Plasmodium falciparum*-infected erythrocytes in the intervillous space of the placenta. This can trigger a local innate inflammatory response called intervillositis. Placental malaria with intervillositis is more strongly associated with LBW than placental malaria without intervillositis [5].

In malaria-endemic regions, up to 70% of LBW cases are due to fetal growth restriction [6] through unknown mechanisms. Pathogenetic pathways suggested to link intervillositis to fetal growth restriction or LBW almost unanimously imply impaired placental development and/or function. For example, the activation of the complement system by placental malaria has been proposed to impair placental vascular development, contributing to poor fetal growth [7] We have shown that placental malaria-associated intervillositis decreases placental amino acid uptake, contributing to fetal growth restriction [8]. How the placenta responds to this relative nutrient deprivation is unknown, but we reasoned that mechanisms that conserve nutrients, such as autophagy, may be invoked.

The placenta is an ideal tissue to study autophagy [9, 10] where it could contribute to the generation of intracellular nutrients [11]. Studies of autophagy in the placenta is a developing field (reviewed in [12, 13]) but there is no published report on placental autophagy in malaria in pregnancy. Macroautophagy, referred to herein as autophagy, is the most prevalent form of autophagy in which intracellular proteins or organelles are sequestered into autophagosomes that fuse with lysosomes to form autophagolysosomes leading to the degradation of the enclosed cargo, the products of which, including free amino acids, are reused by the cell [14]. Autophagosomes are characterized by a double membrane structure, the expansion of which depends on the microtubule-associated protein light chain 3-phosphatidylethanolamine (LC3-PE) system, in which cytosolic LC3-I is covalently bound to PE to generate LC3-II on the autophagosomal membrane [15]. The LC3-II:LC3-I ratio is commonly used as a readout for autophagosome formation whereas lysosome-associated membrane protein 1 (LAMP-1) is a specific marker for lysosomes.

Dysregulation of the physiological autophagic process has been linked to various diseases (reviewed in [16]). Placental autophagy is increased in pregnancies associated with inflammation-induced preterm labor [17], idiopathic fetal growth restriction [18, 19] and preeclampsia [9, 10]. Dysregulated placental autophagy could negatively affect placental function, contributing to LBW. However, the impact of *P*. *falciparum* infection on placental autophagy has not been reported.

Here, we provide unique evidence for dysregulated autophagy in placental malaria with intervillositis that is associated with reduced amino acid uptake by system A and that may contribute to LBW.

## Materials and methods

### Sample collection and selection

The College of Medicine Research Ethics Committee, University of Malawi, approved this study. Written informed consent was obtained from first-time mothers who delivered at the Queen Elizabeth Central Hospital, Blantyre, Malawi. We focused on first-time mothers to avoid any confounding effects from parity on susceptibility to placental malaria, placental malaria-associated intervillositis and the risk of low birthweight. Inclusion and exclusion criteria have been described elsewhere [20]. Placental villous tissue biopsies were collected after delivery. One set was snap-frozen and another was fixed in 10% neutral-buffered formalin and paraffin-embedded. Tissues were grouped based on placental histology into uninfected (no malaria, no intervillositis; n = 17), placental malaria without intervillositis (n = 7), and placental malaria with intervillositis (n = 14). Placental malaria was defined as the presence of infected erythrocytes in the intervillous space. Intervillositis was defined as >5% of the intervillous cells counted being monocytes [8, 21]. In order to assess their histological characteristics, villous tissue biopsies were not washed prior to being frozen or fixed and still contained intervillous blood (~30% v:m). The varying percentage of maternal monocytes between histological groups is however unlikely to significantly impact western blot data. Indeed, in the most severe case of intervillositis in our cohort, maternal monocytes represent 15% of all cells in the intervillous blood. Assuming an haematocrit of 40%, maternal monocytes in this most severe case of intervillositis therefore represent less than 2% of the placental tissue analysed by western blot. As such, the potential contamination from maternal monocytes ranges from 0 to 2% of the total tissue processed. We consider this bias negligible as it is comparable to the imprecisions associated with quantitative western blotting approaches used here. Table 1 summarizes participants’ characteristics. By design, the percentage of monocytes (*P* = .0001) and the placental blood parasitaemia (*P* = .0001) showed differences among groups.

**Table 1:** Characteristics of study subjects.

	Uninfected	Placental malaria without intervillositis	Placental malaria with intervillositis	*P* value
**N**	17	7	14	
**Age, years**	19	18	20	.61
	(18–19)	(17–21)	(18–21)	
**Gestational age, weeks**	40	40	40	.87
	(38–40)	(38–40)	(38–40)	
**Fetal sex, % of females**	44	50	50	>.36*
**Maternal weight at enrolment, kg**	56	56	56	.77
	(49.5–58.5)	(52–60)	(52–56)	
**Percentage of monocytes**	0	2.2	8.6	.0001
		(1.2–3)	(6.6–10.8)	
**Parasitaemia, %**	0	0.41	1.2	.0001
		(0.21–0.83)	(0.66–11.17)	
**Fetal weight, kg**	3	2.8	2.9	.54
	(2.7–3.5)	(2.6–3.1)	(2.6–3.0)	
**Placental weight, g**	500	530	495	.80
	(430–550)	(460–560)	(420–580)	
**Fetal-to-placental weight ratio**	5.95	5.71	5.89	.48
	(5.2–6.48)	(4.11–6.09)	(5.26–6.20)	

Data are median (interquartile range). *P* value of the Kruskal-Wallis test or

*Chi square test.

### Protein extraction from placental homogenate

Placental homogenates were prepared from snap frozen placental villous tissue biopsies. Proteins were extracted using radioimmunoprecipitation assay (RIPA) buffer with protease and phosphatase inhibitor cocktail (Thermo Scientific) and then homogenized using Zirconia beads (Daintree Scientific) on a spiromixer for 30 sec at 4°C, followed by centrifugation at 13,000 x *g* for 15 min at 4°C. Protein concentration was determined using the Lowry assay.

### Western blotting

Placental tissue lysates were prepared in 2X Laemmli buffer. Samples containing 50 μg protein were separated on 4–12% Bis-Tris gels (Invitrogen) and transferred onto 0.45 μm polyvinylidene fluoride membrane (VWR). After blocking with 5% skim milk in Tris-buffered saline (pH 7.6) containing 0.1% Tween-20 (TBS-T), membranes were incubated with primary rabbit-anti-human antibodies against autophagy markers: LC3B (Sigma), p62/SQSTM1 (Sigma), Rab7 (Cell Signaling Technology) and ATG4B (Cell Signaling Technology). Primary antibodies were diluted to 1 μg/ml in TBS-T buffer containing 2% bovine serum albumin. To control for protein loading, the membranes were incubated with mouse anti-β-actin antibody (Sigma) at 0.2 μg/ml. Following washes with TBS-T, the membranes were incubated with horseradish peroxidase-conjugated anti-rabbit or anti-mouse IgG (Cell Signaling Technology). Membranes were visualized by enhanced chemiluminescent substrate (Millipore). Densitometry image analysis was performed using Image J [22].

### Immunofluorescence assay

Tissue sections (3 μm thick) of formalin-fixed, paraffin-embedded placental tissue were mounted onto poly-L-lysine-coated coverslips. Sections were deparaffinised in xylene and rehydrated. Antigen retrieval was performed in 10 mM sodium citrate buffer (pH 7.0) at 95°C for 10 minutes. The sections were then blocked with 5% newborn calf serum (Gibco) in PBS. Three primary antibodies were used: rabbit anti-human LC3B (Sigma), mouse anti-human LAMP1 (deposited to the Developmental Studies Hybridoma Bank by August J. Thomas; DSHB Hybridoma Product 1D4B) and mouse anti-human CK7 (SantaCruz Biotech); a marker of the syncytiotrophoblast. Sections were first incubated with anti-LC3B and anti-LAMP1 antibodies at 10 μg/ml, and then with a secondary antibody mixture of donkey Alexa Fluor 555-conjugated anti-mouse (Molecular Probes) and donkey Alexa Fluor 647-conjugated anti rabbit (Molecular Probes) at 20 μg/ml. After washing, the sections were incubated with anti-CK7 antibodies, followed by incubation with a chicken Alexa Fluor 488-conjugated anti-mouse antibody (Molecular Probes). Sections incubated without primary antibodies were used as negative controls. Sections were mounted in Prolong Gold antifade (Invitrogen) and sealed with nail polish. Confocal microscopy was performed using a LSM 700 microscope (Zeiss) at 100x magnification. Images were captured in 3D (optical thickness of 0.8 μm/slice, four Z steps of 0.5 μm). Alexa Fluor 488, 555 and 647 signals were acquired in separate channels sequentially.

### Image analysis

Imaris software version 7.0 (Bitplane) was used to analyse immunofluorescence images. CK7 stained the syncytiotrophoblast, LAMP1 lysosome puncta and LC3B autophagosome puncta. One microscope field containing terminal villi was chosen as the centre of a matrix of 3x3 immediately adjacent fields automatically generated by the acquisition software. Each field was 200μm x 200μm. All terminal villi within these 9 microscope fields were analysed. The number of terminal villi enumerated per microscope field varied from 3 to 5. The CK7 signal was used to identify the syncytiotrophoblast layer and isolate LC3B and LAMP1 puncta from the syncytiotrophoblast. The number of LC3B and LAMP1 puncta in the syncytiotrophoblast was quantified in tissue sections from five randomly chosen samples from each histological group. Colocalization of LC3B and LAMP1 puncta was expressed as colocalized LC3B/LAMP1 volume, which is the volume of pixels in the syncytiotrophoblast positive for both LC3B and LAMP1. All results were normalized to the volume of the syncytiotrophoblast measured as the volume of CK7 signal. The volume of syncytiotrophoblast analysed was similar across all histological groups (~1,300μm^3^/section; *P* = .085).

### System A activity

^14^C-MeAIB (PerkinElmer) was used as a well-characterized amino acid analogue substrate to measure the activity of system A amino acid transporter [23]. Microvillous plasma membrane (MVM) vesicles were isolated and purified as we described previously [8] from the same placentas as those assessed for the expression of autophagic markers. ^14^C-MeAIB uptake by MVM vesicles was measured as described previously [23]. Na^+^-dependent uptake of ^14^C-MeAIB into MVM vesicles at 30 s was taken to provide an estimate of initial rate [23, 24].

### Statistical analysis

Data are presented as medians and interquartile range. Statistical analyses were performed using Stata 12 software (StataCorp, College Station, Texas). Given the non-normal distribution of the data (established using the Shapiro-Wilk test), two-group comparisons were made using the Mann-Whitney test and three-group comparisons using the Kruskal-Wallis test. Correlations were assessed using Spearman’s correlation test. Multivariate linear regression was conducted to test if parasitaemia and the severity of intervillositis (measured as the percentage of monocytes in the intervillous blood space) both treated as continuous variables predicted the levels of the various markers measured.

Predictive models were developed using log-transformed data (density of colocalized LC3B/LAMP1, density of LC3B and density of LAMP1 were log-transformed) and linear regression models. Model fit was assessed using R-squared statistic, checking for linearity and visualising residuals. Repeated measures from the same individual were accounted for by relaxing the assumption of independence amongst measurements within the same individual by using the stata vce (cluster clustvar) command.

## Results

### Higher number of autophagosomes in placental malaria with intervillositis

**Fig 1:**
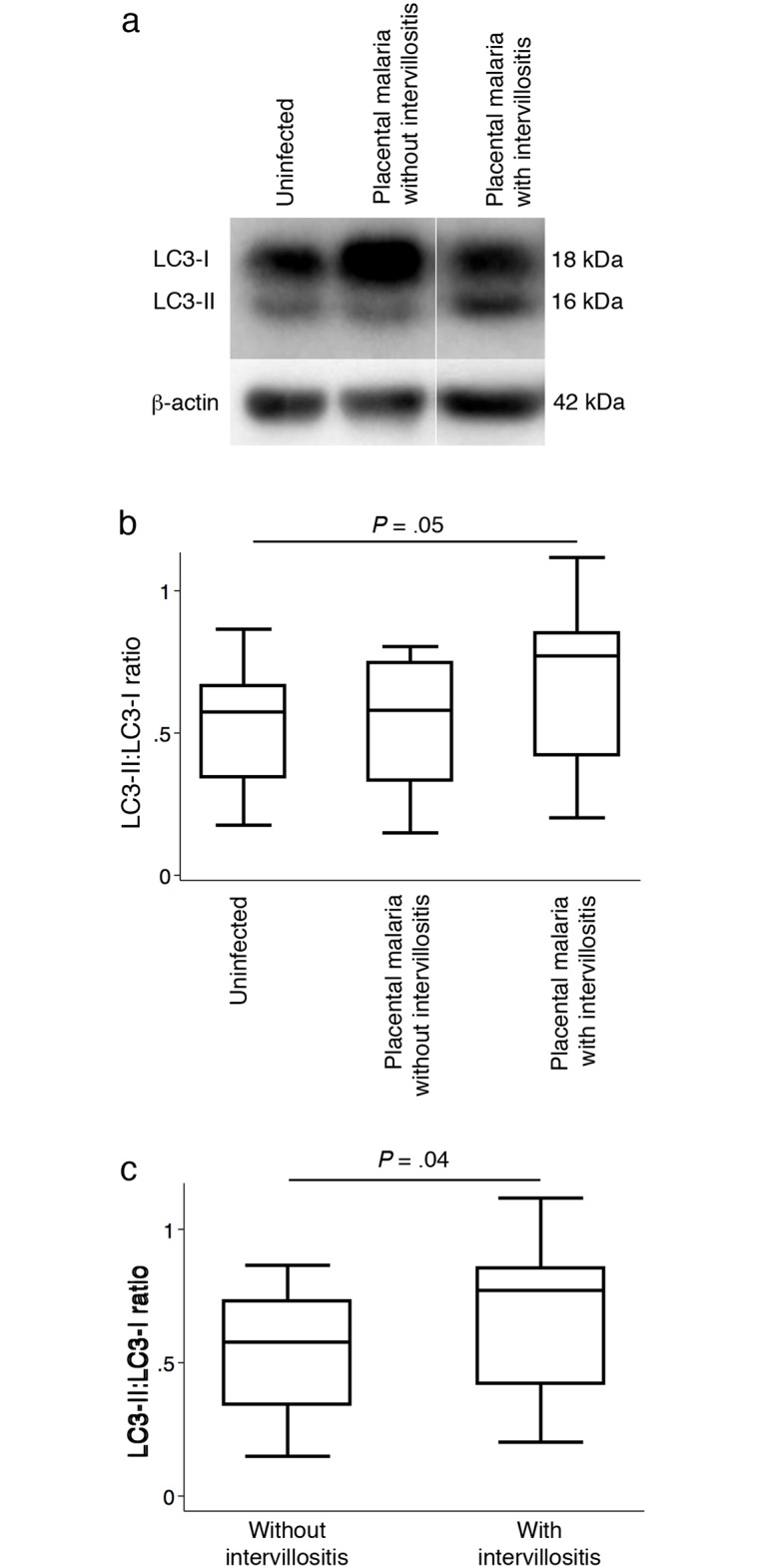
Autophagosome formation in placental homogenates. (A) Representative LC3 western blot of uninfected placenta, placental malaria without intervillositis and placental malaria with intervillositis. Densitometry analysis of LC3-II:LC3-I ratio (B) in uninfected placentas (n = 17) and infected placentas without (n = 7) or with (n = 14) intervillositis and (C) between placentas without (n = 24) and with (n = 14) intervillositis. Data are presented as box plots. Bars represent median, 25^th^ and 75^th^ percentiles and the whiskers are 5^th^ and 95^th^ percentiles.

The ratio of LC3-II to LC3-I protein levels in placental homogenates was used as the readout for the number of autophagosomes present (Fig 1A). The LC3-II:LC3-I ratio was higher in placental malaria with intervillositis compared with uninfected controls (*P* = .05) (Fig 1B). There was no significant difference in LC3-II:LC3-I ratio between uninfected controls and placental malaria without intervillositis (*P* = .82). The importance of intervillositis in driving the higher LC3-II:LC3-I ratio was further evidenced by the higher (*P* = .04) LC3-II:LC3-I ratio in placentas with intervillositis compared to placentas without intervillositis, regardless of their infection status (Fig 1C) as well as by the positive correlation (n = 38; R = 0.33; *P* = .04) between LC3-II:LC3-I ratio and the severity of intervillositis measured as the percentage of monocytes in the intervillous blood space. Using multivariate linear regression analysis, we found that the percentage of monocytes in the intervillous spaces and parasitaemia explain a significant amount of the variance in LC3-II:LC3-I ratio (F(2,30) = 25.62; *P* < .0001; R^2^ = .63). The percentage of monocytes in the intervillous spaces significantly predicted LC3-II:LC3-I ratio (ß = .057; t(32) = 6.56; *P* < .0001) but parasitaemia did not (ß = .0006; t(32) = 0.66; *P* = .52). These results suggest that the inflammatory response induced by placental malaria is associated with a higher number of autophagosomes.

### Higher density of autophagosomes and lysosomes in placental malaria with intervillositis

**Fig 2:**
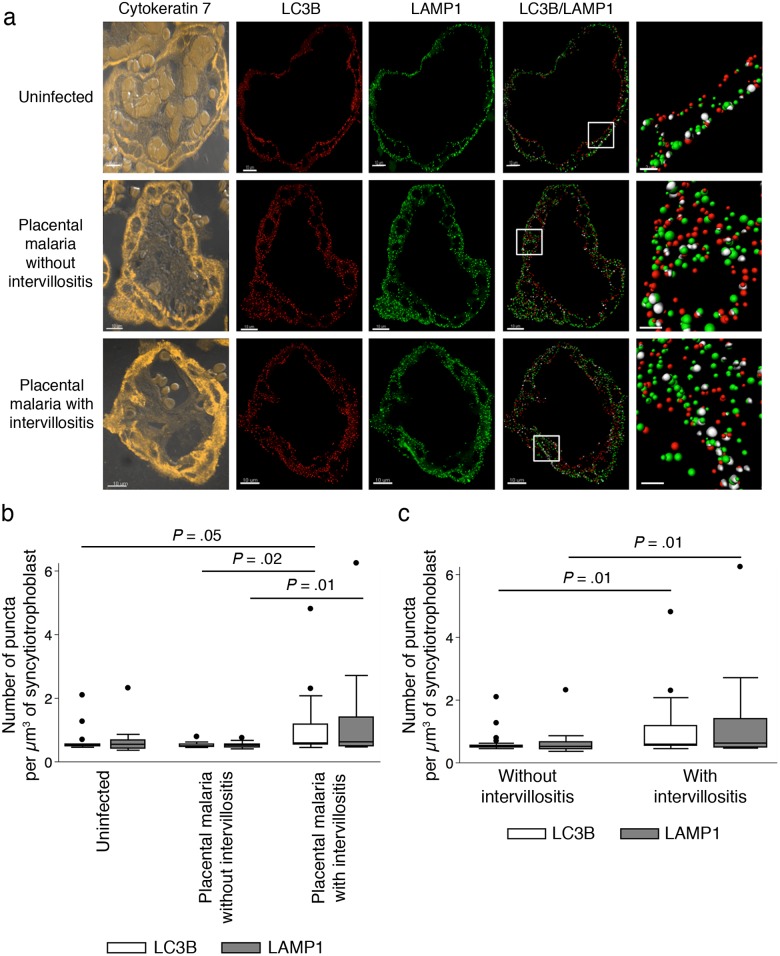
Analysis of the density of autophagosomes and lysosomes in syncytiotrophoblasts. (A) Cytokeratin 7 (in yellow) was used to identify the syncytiotrophoblast. LC3B puncta (in red) identified autophagosomes. LAMP1 puncta (in green) identified lysosomes. Their colocalization is shown in white in the merged images. Scale bar: 10μm, except for colocalization images: 2μm. The density of autophagosomes and lysosomes normalised to the volume of syncytiotrophoblast was compared (B) across placental histology groups and (C) between placentas with, or without, intervillositis. Data were acquired from terminal villi on tissue sections from five randomly chosen samples from each histological group (nine microscope fields per section). Data are presented as box plots. Bars represent median, 25^th^ and 75^th^ percentiles, the whiskers are 5^th^ and 95^th^ percentiles and the dots are outliers.

We investigated if the higher LC3-II:LC3-I ratio observed in placental malaria with intervillositis was associated with a higher density of autophagosomes (as well as lysosomes/late endosomes). Using immunofluorescence and advanced 3D image analysis, we quantified the density of LC3B puncta (identifying autophagosomes) and LAMP1 puncta (identifying lysosomes) in the syncytiotrophoblast (Fig 2A). Our image analysis approach was validated by the positive correlation (n = 15; R = .44; *P* = .003) between the density of LC3B puncta measured by immunofluorescence and the LC3-II:LC3-I ratio measured by western blot.

The density of LC3B puncta (Fig 2B) was higher in placental malaria with intervillositis compared with uninfected controls (*P* = .05), or placental malaria without intervillositis (*P* = .02). The density of LAMP1 puncta (Fig 2B) was also higher in placental malaria with intervillositis than in placental malaria without intervillositis (*P* = .01), or than in uninfected controls (*P* = .09). There was no significant difference in the density of LC3 puncta, or LAMP1 puncta, between uninfected controls and placental malaria without intervillositis (*P* ≥ .10). The higher parasitaemia in the group with placental malaria with intervillositis compared to the group with placental malaria without intervillositis did not confound these differences as parasitaemia correlated neither with the density of LC3B puncta (n = 15; R = 0.003; *P* = .45), nor with that of LAMP1 puncta (n = 15; R = 0.003; *P* = .56). When the samples were categorised based on the presence of intervillositis regardless of infection status, placentas with intervillositis showed higher densities of both LC3B (*P* = .01) and LAMP1 (*P* = .01) puncta compared to placentas without intervillositis (Fig 2C). Using multivariate linear regression analysis, we found that the percentage of monocytes in the intervillous spaces and parasitaemia explain a significant amount of the variance in the density of LC3B puncta (F(2,30) = 3.12; *P* = .049; R^2^ = .17) and LAMP1 puncta (F(2,30) = 2.7; *P* = .043; R^2^ = .15). The percentage of monocytes in the intervillous spaces significantly predicted the density of LC3B puncta (ß = .081; t(32) = 2.35; *P* = .026) and LAMP1 puncta (ß = .102; t(32) = 2.24; *P* = .033) but parasitaemia did not (LC3B puncta: ß = .00025; t(32) = 0.07; *P* = .95; LAMP1 puncta: ß = -0.0006; t(32) = -0.13; *P* = .9). These findings suggest higher density of autophagosomes and lysosomes in the syncytiotrophoblast of women with placental malaria with intervillositis.

### Increase in LC3B/LAMP1 colocalization in placental malaria with intervillositis largely explained by forced encounters

**Fig 3:**
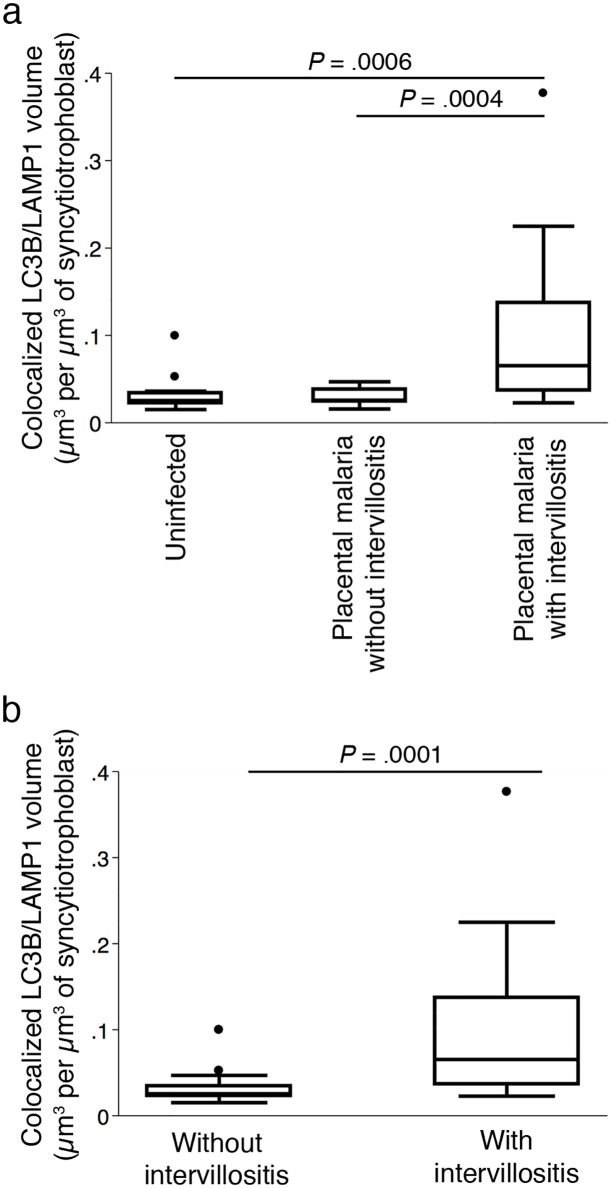
LC3B and LAMP1 puncta colocalization measured as the volume of colocalized LC3B/LAMP1 signals. Volume of colocalized LC3B/LAMP1 signals per μm^3^ of syncytiotrophoblast (A) across the three placental histology groups and (B) between placentas without and with intervillositis. Data were acquired from terminal villi on tissue sections from five randomly chosen samples from each histological group (nine microscope fields per section). Data are presented as box plots. Bars represent median, 25^th^ and 75^th^ percentiles, the whiskers are 5^th^ and 95^th^ percentiles and the dots are outliers.

We next investigated whether the higher density of autophagosomes and lysosomes observed in the syncytiotrophoblast of women with placental malaria with intervillositis translated into increased autophagosome/lysosome fusion. We measured LC3B/LAMP1 colocalized volume as a quantitative indicator of autophagosomes/lysosome fusion (Fig 3A). LC3B/LAMP1 colocalized volume was higher in placental malaria with intervillositis than in uninfected placentas (*P* = .0006), or placental malaria without intervillositis (*P* = .0004) (Fig 3A). There was no significant difference in LC3B/LAMP1 colocalized volume between uninfected controls and placental malaria without intervillositis (*P* = .82). The higher parasitaemia in placental malaria with intervillositis compared to placental malaria without intervillositis did not confound this difference as parasitaemia did not correlate with the LC3B/LAMP1 colocalized volume (n = 15; R = 8.14; *P* = .64). When the samples were categorised based on the presence of intervillositis regardless of infection status, placentas with intervillositis showed higher LC3B/LAMP1 colocalized volume compared to placentas without intervillositis (*P* = .0001) (Fig 3B). LC3B/LAMP1 colocalized volume was positively correlated with the percentage of monocytes in the intervillous blood space (n = 15; R = .41; *P* = .009), but not with intervillous blood parasitaemia (n = 15; R = .25; *P* = .11). Using multivariate linear regression analysis, we found that the percentage of monocytes in the intervillous spaces and parasitaemia explain a significant amount of the variance in the LC3B/LAMP1 colocalized volume (F(2,36) = 3.12; *P* = .046; R^2^ = .15). The percentage of monocytes in the intervillous spaces significantly predicted the LC3B/LAMP1 colocalized volume (ß = .032; t(38) = 2.45; *P* = .019) but parasitaemia did not (ß = -0.0006; t(38) = -0.37; *P* = .71). These findings could suggest that the inflammatory response to placental malaria promotes autophagolysosome formation.

However, the higher LC3B/LAMP1 colocalized volume in placental malaria with intervillositis could also be due, at least in part, to the higher density of LC3B and LAMP1 puncta in these placentas (Fig 2). Predictive models revealed that about 85% of the increase in LC3B/LAMP1 colocalized volume measured in placental malaria with intervillositis could be explained the higher density of autophagosomes and lysosomes measured in this group that caused forced encounters between autophagosomes and lysosomes (Univariate R^2^: .84 for LC3B and .89 for LAMP1). These models suggested that only ~15% of the higher LC3B/LAMP1 colocalized volume in placental malaria with intervillositis is attributable to increased autophagic process. These data suggest that a very large proportion of the higher LC3B/LAMP1 colocalized volume in placental malaria with intervillositis may not be the result of an increase in autophagic process and may therefore not be biologically relevant and alternatively might indicate a block in autophagosome/lysosome fusion.

### A possible block in autophagosome/lysosome fusion in placental malaria with intervillositis

**Fig 4:**
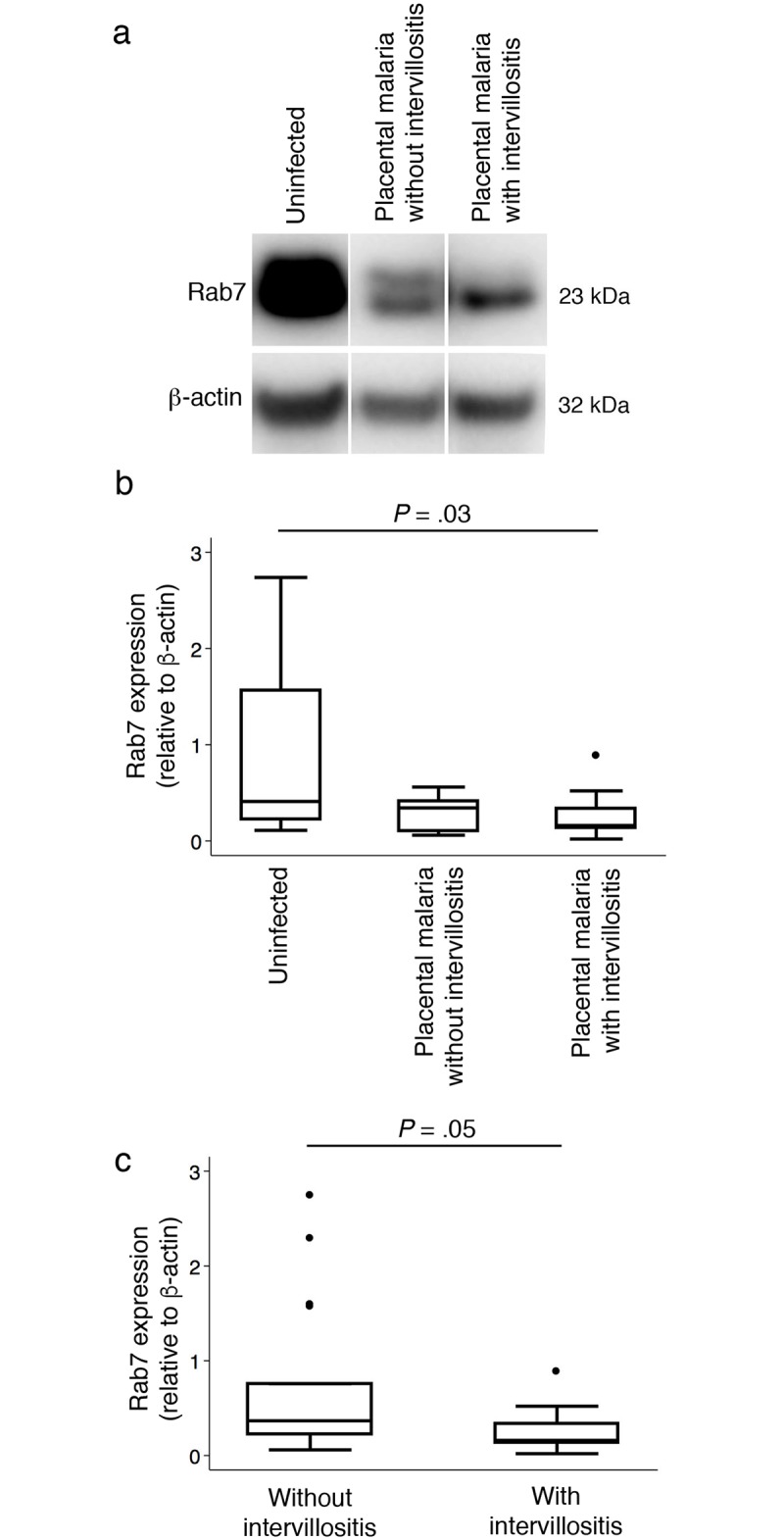
Expression of Rab7 in placental homogenates. (A) Representative western blot of uninfected, placental malaria without intervillositis and placental malaria with intervillositis. Rab7 expression was compared (B) between uninfected placentas (n = 17) and infected placentas without (n = 7) or with (n = 14) intervillositis and (C) between placentas without (n = 24) and with (n = 14) intervillositis. Bars represent median, 25^th^ and 75^th^ percentiles, the whiskers are 5^th^ and 95^th^ percentiles and the dots are outliers.

The increased density of autophagosomes and lysosomes and the apparent absence of biologically relevant increase in autophagolysosome formation were indicative of a block in autophagosome/lysosome fusion in placental malaria with intervillositis. In order to test this hypothesis, we investigated the expression of Rab7 (Fig 4A), a small GTPase member of the RAS oncogene family functioning in multiple biological processes (for review see [25]) including being a key mediator of autophagosome/lysosome fusion and of lysosome degradation. Depletion of Rab7 leads to a block in autophagosome/lysosome fusion and prevents lysosomal degradation [26, 27].

Rab7 expression was lower in placental malaria with intervillositis than in uninfected placentas (*P* = .03) (Fig 4B). The higher parasitaemia in the group with placental malaria with intervillositis compared to the group with placental malaria without intervillositis did not confound this difference as parasitaemia did not correlate with Rab7 expression (n = 21; R = -4.97; *P* = .45). Placentas with intervillositis had lower Rab7 levels compared to placentas without intervillositis (*P* = .05) regardless of their infection status (Fig 4C). Furthermore, Rab7 levels were negatively correlated with the percentage of monocytes in the intervillous blood space (n = 38; R = -.36; *P* = .04). Using multivariate linear regression analysis, we found that the percentage of monocytes in the intervillous spaces and parasitaemia explain a significant amount of the variance in Rab7 levels (F(2,30) = 7.1; *P* = .003; R^2^ = .32). The percentage of monocytes in the intervillous spaces significantly predicted Rab7 levels (ß = -0.012; t(32) = -2.63; *P* = .013) but parasitaemia did not (ß = -0.0024; t(32) = -0.65; *P* = .52).

In uninfected placentas, Rab7 expression and the density of LAMP1 puncta were strongly positively correlated (n = 5; R = .95; *P* = .0001). This association was totally reversed to a strong negative correlation in infected placentas (n = 10; R = -.59; *P* = .0006), which was found in placentas with intervillositis (n = 5; R = -.53; *P* = .04), but not in placentas without intervillositis (n = 10; R = -.21; *P* = .33). These findings further support our model that the inflammatory response to placental malaria is associated with a block in autophagosome/lysosome fusion.

**Fig 5:**
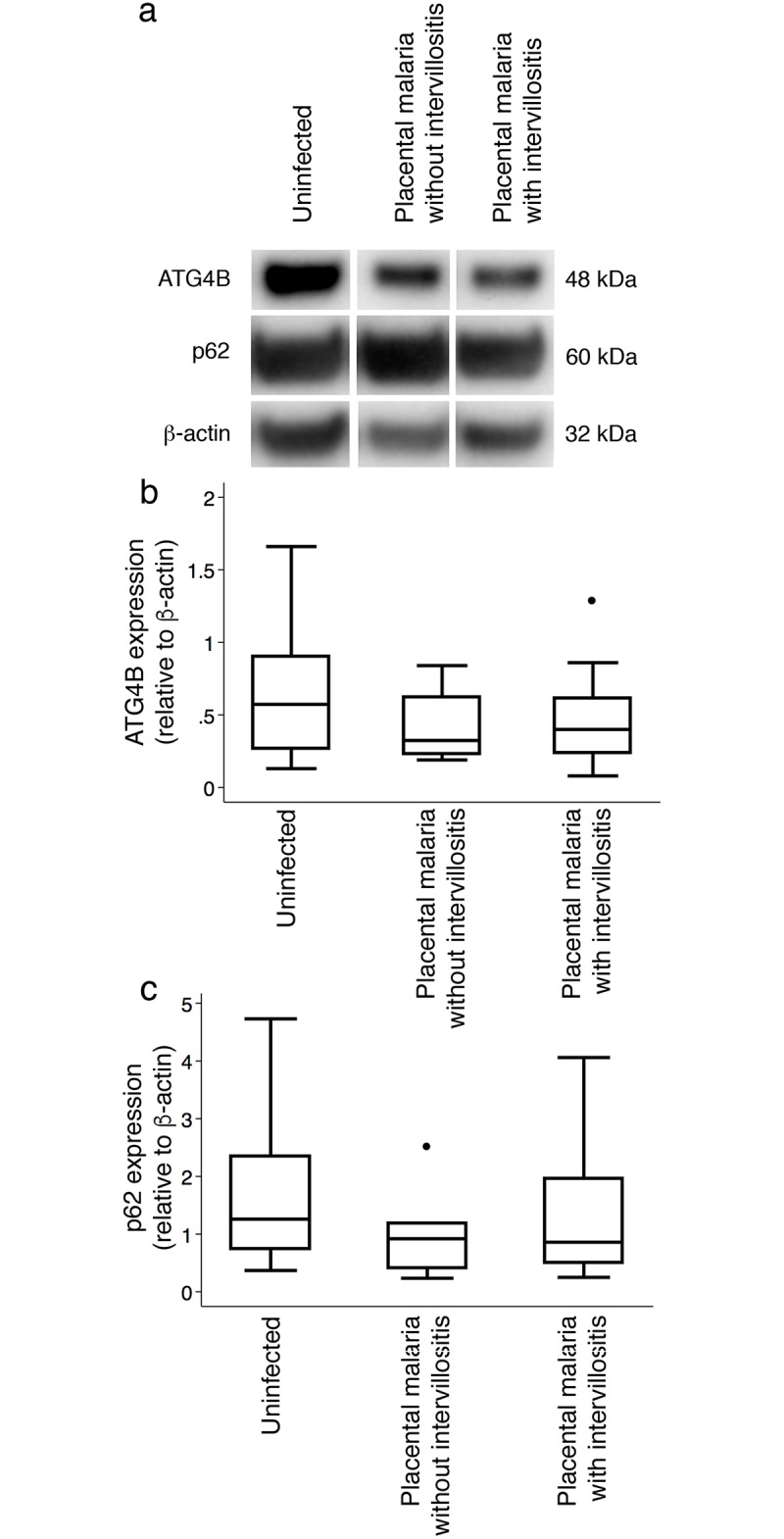
Expression of ATG4B and p62 in placental homogenates. (A) Representative western blots of uninfected, placental malaria without intervillositis (PM without IV) and placental malaria with intervillositis (PM with IV). (B) ATG4B and (C) p62 expression (relative to β-actin) were compared between uninfected placentas (n = 17) and infected placentas without (n = 7) or with (n = 14) intervillositis. Bars represent median, 25^th^ and 75^th^ percentiles, the whiskers are 5^th^ and 95^th^ percentiles and the dots are outliers.

To further examine autophagosome maturation, we determined the expression of ATG4B and of SQSTM1/p62 (Fig 5A). ATG4B is a cysteine protease key to autophagosome processing by the activation of the LC3 precursor to LC3-II, and also through the delipidation of LC3-II from autophagosome outer membranes for recycling [28]. ATG4B expression (Fig 5B) showed no significant differences across histological groups (*P* = .32), suggesting normal autophagosome maturation. SQSTM1/p62 mediates the incorporation of ubiquitinated protein aggregates into autophagosomes that are then cleared during maturation into autophagolysosomes [29]. In a normal autophagic process, p62 is itself degraded during autophagy. We found no significant difference in p62 expression across histological groups (*P* = .15) suggesting that the loading of cargo into autophagosomes occurs normally in all histological groups (Fig 5C). The similar levels of p62 across histological groups despite higher autophagosome formation in placental malaria with intervillositis (indicated by the higher LC3-II:LC3-I ratio) are indicative of impaired autophagy, downstream of p62 recruitment in these placentas.

### The accumulation of autophagosomes and lysosomes is associated with decreased amino acid uptake

Fetal nutrient supply, especially amino acids, is the single strongest determinant of fetal growth (and therefore birthweight) [24] and largely depends on the amino acid transport capacity of the placenta. System A is a group of amino acid transporters critical for placental transport of both non-essential and essential amino acids. We first showed that placental malaria-associated intervillositis reduced System A activity, which positively correlated with birthweight [8]. Moreover, System A activity negatively correlates with the severity of fetal growth restriction in humans [24].

To determine whether the block in autophagosome/lysosome fusion we identified in the syncytiotrophoblast of malaria-infected placentas with intervillositis was associated with decreased amino acid uptake, we assessed the correlation between autophagy and System A activity. System A activity correlated negatively with the LC3-II:LC3-I ratio (n = 38; R = -.51; *P* = .001) and the densities of LC3 puncta (n = 15; R = -.3; *P* = .06) and LAMP1 puncta (n = 15; R = -.42; *P* = .008). In uninfected placentas (n = 5), fetal birthweight correlated negatively with the densities of LC3 puncta (R = -.48; *P* = .073) and LAMP1 puncta (R = -.67; *P* = .0061). These correlations were not found in infected placentas (n = 10; *P* ≥ .22).

Collectively, these findings provide strong evidence that the inflammatory response to placental malaria is associated with dysregulation in the late stages of the autophagy pathway and that this could impair placental transplacental amino acid transport, contributing to poor fetal growth.

## Discussion

We provide the first evidence for dysfunctional autophagy in placental malaria, which appears to be largely driven by the inflammatory response to placental malaria. The locus of autophagic dysfunction was evidenced as a block in autophagosome/lysosome fusion, which could impair amino acid uptake and contribute to poor fetal growth.

Our study uniquely reports the impact of a defined maternal infection on the autophagic process in the placenta. Placental autophagy has been studied in non-infectious pregnancy complications such as inflammation-induced preterm labour [17], idiopathic fetal growth restriction [18, 19] and preeclampsia [9, 10]. However, these studies have mostly reported increased autophagosome formation and very few addressed downstream events in the autophagic process, or addressed the pathogenic role of placental autophagy. Yet, increased placental autophagy could be either protective or detrimental, probably based on the metabolic status of the cell or tissue [30]. Here, we used a multi-technique and multi-angle approach to address various steps of the autophagic process and investigated the role for dysregulated autophagy in the pathogenesis of fetal growth restriction associated with malaria in pregnancy.

As reported for other non-infectious pregnancy complications [9, 17–19], we first observed increased autophagosome formation in placental malaria with intervillositis. This was confirmed by the higher density of autophagosomes in the syncytiotrophoblast of infected placentas with intervillositis. Further, we investigated subsequent steps in the autophagic process and evaluated readouts of autophagic activity [31]. We found a higher density of lysosomes in the syncytiotrophoblast of infected placentas with intervillositis that paralleled with a higher colocalization of LC3B/LAMP1 (readout for autophagolysosome formation) in this histological group. However, predictive models established that the vast majority of these colocalization events between autophagosomes and lysosomes happen by chance and are forced encounters, probably due to the higher density of autophagosomes and lysosomes in the syncytiotrophoblast of infected placentas with intervillositis. These results indicated that the increased autophagosome and lysosome densities did not translate into the formation of autophagolysosomes necessary for the degradation and recycling of the autophagosome cargo, which would therefore accumulate in the syncytiotrophoblast of infected placentas with intervillositis, with possible toxic consequences for the tissue [32].

**Fig 6:**
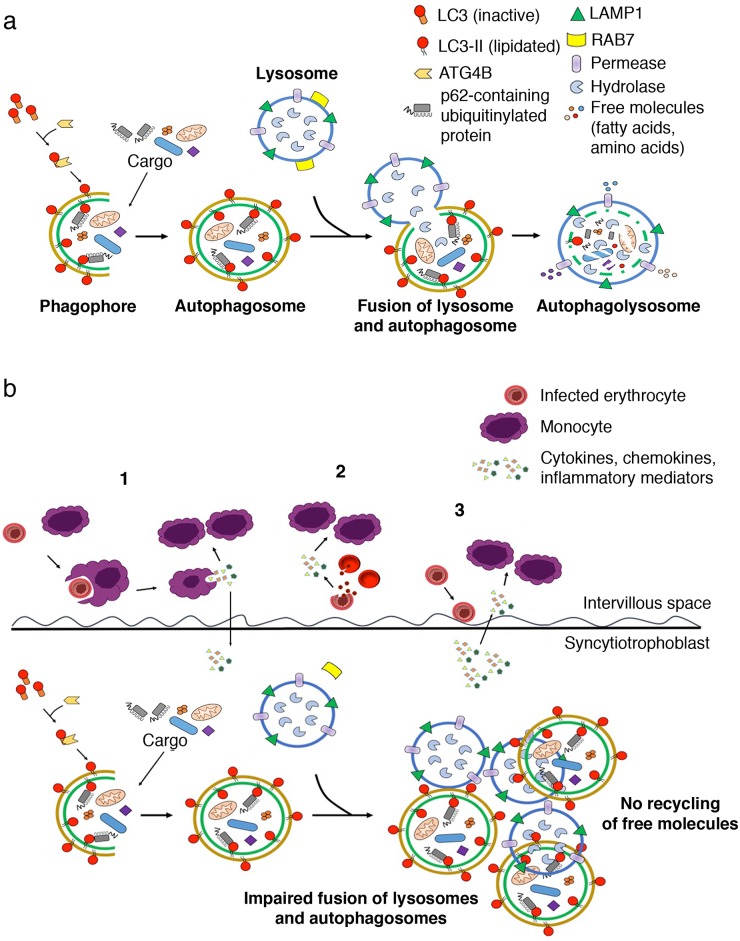
Proposed mechanisms of autophagy in placental malaria with intervillositis. (A) Normal autophagic process leading to the recycling of free molecules, including fatty acids and amino acids. (B) In placental malaria with intervillositis, cytokines, chemokines and other inflammatory mediators are released (**1**) by monocytes after phagocytosis of *P*. *falciparum*-infected erythrocytes, (**2**) following the rupture of *P*. *falciparum*-infected erythrocytes and (**3**) by the syncytiotrophoblast in response to the adhesion of *P*. *falciparum*-infected erythrocytes. This inflammatory response is associated with impaired autophagosome/lysosome fusion that leads to an accumulation of autophagosomes and lysosomes in the syncytiotrophoblast, which we propose contributes to impaired transplacental amino acid transport, leading to poor fetal growth and low birthweight.

Our results indicate that autophagosomes and lysosomes accumulate in the syncytiotrophoblast of infected placentas with intervillositis due to a block in autophagosome/lysosome fusion. This fusion process is highly regulated [33] and requires Rab7 [34, 35]. The lower Rab7 expression in these placentas supports our hypothesis for a block in autophagosome/lysosome fusion in placental malaria with intervillositis. The anti-Rab7 antibody used in these studies allows assessment of the total level of Rab7 protein. Importantly, as a GTPase, Rab7 protein undergoes cyclical activation and inactivation depending on GTP binding and hydrolysis; the active state being that where GTP is bound [25]. As such, the activity of Rab7 may be more important in regulating autophagosome/lysosome fusion than Rab7 expression levels [36]. Rab7 activity is positively regulated by insulin-like growth factor-1 (IGF-I) [37]. We have previously reported lower circulating levels of IGF-I in placental malaria with intervillositis [38]. It is therefore possible that low IGF-I levels in placental malaria with intervillositis could inhibit Rab7 activity, contributing to a block in autophagosome/lysosome fusion. This is supported by the negative correlation between Rab7 expression levels and the density of lysosomes in placental malaria with intervillositis and suggests defective lysosomal function that could result in impaired autophagosome/lysosome fusion and subsequently, impaired cargo degradation [39]. Taken together, our results suggest that autophagosome/lysosome fusion is dysfunctional in placental malaria with intervillositis (Fig 6). A similar block in autophagosome/lysosome fusion has been involved in the pathogenesis of lysosomal storage diseases [40], Alzheimer’s disease [41] and has been described in human epithelial cells infected by influenza A virus [42]. However, it had never been reported in the placenta.

Our findings that dysregulated autophagy appears to be specifically associated with the inflammatory response to malaria rather than infection itself is in keeping with the well-established bi-directional link between autophagy and inflammation [43]. Pro-inflammatory cytokines, including IL-1ß and TNF-α, have been shown to induce autophagy [43]. These cytokines are found at elevated levels in placental malaria with intervillositis and contribute to the pathogenesis of poor fetal growth [8, 21, 44]. Conversely, dysfunctional autophagy could activate inflammatory pathways and the production of pro-inflammatory mediators [17]. The dysfunctional autophagic process in placental malaria-associated intervillositis could contribute to the local inflammatory milieu, which would impair nutrient transport and compromise fetal growth [8, 45]. This link between dysfunctional autophagy and compromised fetal growth is supported by the negative correlation we observed between the density of autophagosomes and lysosomes and amino acid uptake; a major regulator of fetal growth in humans [24]. Mechanistic target of rapamycin (mTOR) is an important negative regulator of autophagy [46] and a positive regulator of placental amino acid uptake [47]. We have recently shown that the inflammatory response to placental malaria inhibits placental mTOR signalling, reducing placental amino acid uptake, leading to fetal growth restriction [48]. mTOR inhibition triggered by the inflammatory response to malaria could be responsible for the increased autophagosome formation in placental malaria with intervillositis, as evidenced by the higher LC3-II:LC3-I ratio in these placentas. However, our data suggest that placental malaria with intervillositis impairs autophagosome/lysosome fusion, resulting in dysregulated autophagic flux. We propose that this dysregulated autophagic flux could contribute increased pathology as it has been described in aging, Parkinson’s disease, and other neuro-and myodegenerative disorders (reviewed in [47]). In particular, altered autophagic flux could enhance inflammation [17], a key trigger for decreased nutrient transport and impaired fetal growth [8, 48, 49]. This suggests a pathogenic role for placental autophagy in placental malaria. This is reinforced by the findings that the dysregulation of autophagy is more pronounced in placental malaria with intervillositis, a condition linked with worse pregnancy outcomes than placental infection on its own [21]. Given the complex and bi-directional link between autophagy and inflammation, further studies are needed to examine the relationship between dysregulated autophagy and LBW.

Our findings open up new avenues of research into therapeutics or strategies to optimise fetal growth in malaria-exposed women by improving the placental autophagic process, which could restore fetal amino acid supply. For maximised efficacy, such approaches would be used in combination with established anti-malarial therapies and preventative interventions. Interventions to stimulate autophagy have been trialled in various diseases [50] and nanoparticle-based drug formulations could have the potential to selectively target the placenta [51]. However, we found no evidence for a defect in the initial phases of the autophagic process. Instead, the dysregulation identified here appears to specifically involve a block in autophagosome/lysosome fusion. Any approach should specifically aim at correcting this fusion defect.

In summary, about 85 million women are at risk of malaria in pregnancy, resulting in ~900,000 LBW deliveries every year [52]. Despite all the interventions available, 25% of women have evidence of placental malaria in Sub-Saharan Africa where it causes up to 14% of all LBW deliveries [4]. Because LBW increases the risk of neonatal and infant mortality and morbidity, there is an urgent need to identify the mechanisms linking placental malaria to LBW in order to propose interventions aimed at improving infant health by increasing birthweight. Here, we provide unique demonstration of the detrimental impact of the placental inflammatory response to malaria infection on placental autophagy. Furthermore, this is the first report on the impact of any defined maternal infection on the human placental autophagic process. We provide evidence that placental malaria-associated intervillositis is associated with impaired autophagosome/lysosome fusion. This dysregulated autophagy was associated with impaired transplacental amino acid transport, which could contribute to poor fetal growth and LBW. Future research aimed at restoring an adequate autophagic response to placental malaria or other insults, or preventing autophagic dysfunction, is a priority.

## Abbreviations

ATG4B: Autophagy Related 4B Cysteine Peptidase
CK7: cytokeratin 7
Ig: immunoglobulin
IGF-I: insulin-like growth factor-1
LAMP-1: lysosome-associated membrane protein 1
LBW: low birthweight
LC3: microtubule-associated protein light chain 3
MeAIB: methyl alpha-aminoisobutyric acid
MVM: Microvillous plasma membrane
p62/SQSTM1: ubiquitin-binding protein/sequestosome-1
PBS: phosphate buffer saline
PE: phosphatidylethanolamine
Rab7: Ras-related protein 7
RIPA: radioimmunoprecipitation assay
TBS: Tris-buffered saline
UN: United Nations
WHO: World Health Organization
